# Development and content validity of an AI-assisted simulation scenario for orthotic fabrication in occupational therapy education

**DOI:** 10.1186/s12909-026-10043-x

**Published:** 2026-07-29

**Authors:** Duygu Mine Alataş, Çiğdem Öksüz, Nilgün Bek, Özlem Ülkü Bulut, Kamile Akarsu, Mevlüt Karadağ, Aynur Ayşe Karaduman

**Affiliations:** 1 Department of Occupational Therapy, Faculty of Health Sciences, Lokman Hekim University, Söğütözü Mh. 2185 Cd. No: 20/J, Çankaya, Ankara, 06510 Türkiye; 2 Department of Occupational Therapy, Faculty of Health Sciences, Hacettepe University, Ankara, Türkiye; 3 Department of Physiotherapy and Rehabilitation, Faculty of Health Sciences, Lokman Hekim University, Ankara, Türkiye; 4 Department of Obstetric and Gynecology Nursing, Faculty of Health Sciences, Lokman Hekim University, Ankara, Türkiye; 5 Department of Surgical Nursing, Faculty of Health Sciences, Lokman Hekim University, Ankara, Türkiye; 6 Department of Health Services Management, Faculty of Health Sciences, Lokman Hekim University, Ankara, Türkiye

**Keywords:** Orthotic devices, Active learning, Large language models, Competency, Simulation training

## Abstract

**Background:**

Developing high-fidelity simulation scenarios is a time-consuming process, and validated resources for complex psychomotor skills such as orthotic fabrication are scarce. This study aimed to examine the content validity index (CVI) of a simulation scenario for anticlaw orthosis fabrication developed with the support of artificial intelligence (AI).

**Methods:**

A mixed-methods methodological study was conducted in an academic education program in Türkiye. Six subject matter experts with professional experience in hand rehabilitation, orthotics, and simulation-based education participated. A simulation scenario package was structured by the researchers, while the specific scenario flow was generated using a seven-step process supported by the generative AI tool ChatGPT. Expert reviewers evaluated five components, specifically objectives, background, prebriefing, scenario flow, and debriefing, using a 4-point Likert scale and open-ended questions. The item-level content validity index (I-CVI) and scale level content validity index (S-CVI) were calculated.

**Results:**

The simulation scenario package was evaluated across five core components. The scenario achieved an I-CVI and S-CVI of 1.00. This indicated that all expert reviewers rated the scenario components as content valid by assigning a score of 3 or 4. Qualitative feedback highlighted the need for refinements in clinical fidelity, specific material choices, and safety warnings regarding heat and sharp tools.

**Conclusions:**

The study provided educators with a comprehensive, ready-to-implement simulation scenario package, including validated learning objectives, prebriefing instructions, the full scenario script developed via AI-assisted methodology, and debriefing guides, that could be adopted to enhance orthotic training in rehabilitation curricula.

**Supplementary Information:**

The online version contains supplementary material available at https://doi.org/10.1186/s12909-026-10043-x.

## Background

Simulation has long served as a foundational educational strategy, with its origins rooted in high risk fields such as aviation [1]. Simulation based applications function as a bridge between theoretical knowledge and real world practice [2]. In healthcare education, the use of controlled and simulated environments enables students to develop clinical skills in a risk free setting, thereby enhancing confidence and supporting the integration of theoretical knowledge with practical application [3, 4]. Accordingly, simulation based education supports the development of learners’ knowledge, skills, and professional attitudes, while contributing to patient safety by minimizing unnecessary risks [5].

Within occupational therapy curricula, orthotic fabrication represents a particularly complex learning domain. This area requires the integration of anatomical, biomechanical, and physiological knowledge alongside the development of advanced psychomotor skills and strict adherence to safety principles [6, 7]. Traditionally, orthotic fabrication has been taught through instructor led demonstrations in laboratory settings [8]. However, such approaches may be limited in their ability to foster essential twenty first century competencies, including autonomy, problem solving, and critical thinking, and may not consistently provide the structured conditions necessary to maximize experiential learning [9]. In contrast, simulation based learning has been shown to promote active engagement and reflective thinking among students [10].

Although the use of simulation in occupational therapy education has increased over time [11, 12], its application specifically in orthotic fabrication training remains limited. With the exception of one study utilising artificial hand models to support skill acquisition [13], there is a notable lack of structured simulation scenarios that reflect authentic clinical encounters [14]. The effectiveness of simulation scenarios is contingent upon their level of fidelity, defined as the extent to which they mirror clinical reality [15]. Developing high fidelity scenarios, however, requires substantial time, clinical expertise, and pedagogical experience, which may place a considerable burden on educators [16].

Recent advances in artificial intelligence (AI) have drawn attention to the potential use of AI supported tools to assist with educational content development, including simulation scenario design [17]. However, the use of simulation scenarios in education or research is contingent upon their validity [18]. Without established content validity, simulation activities may fail to meet learning objectives or may lead to inaccurate or unsafe educational outcomes [19]. Accordingly, AI assisted educational materials must be subject to the same rigorous development and validation processes as traditionally developed resources.

In this context, simulation scenarios designed for orthotic fabrication must accurately reflect clinical realities, given the technical demands and patient safety considerations inherent to this area of practice. At the same time, there is a need for approaches that can support educators in managing the time and expertise required for scenario development. The integration of large language model (LLM) tools such as ChatGPT has increasingly been described as an educational imperative that requires careful attention to safety, ethics, and professional standards [20]. Responding to this need, the present study examines the content validity of a simulation scenario for anticlaw orthosis fabrication developed with the support of ChatGPT. By prioritizing evaluation expert reviewers and structured validation processes over direct student exposure, this study seeks to evaluate the applicability of established simulation development and validation standards to AI assisted educational materials within occupational therapy education. Therefore, the aim of this study was to develop and validate a high fidelity simulation scenario for anticlaw orthosis fabrication. Beyond validating the content, this study seeks to offer the academic community a comprehensive simulation scenario package. By providing validated learning objectives, prebriefing protocols, the full scenario script, and debriefing guides, we aim to equip educators with a refined and adaptable resource that ensures clinical competency and patient safety in orthotic education.

## Methods

### Study design

The present study employed a mixed methods design to examine the content validity of a simulation scenario developed with the support of generative AI. The validity of the scenario was assessed quantitatively through the calculation of the content validity index (CVI), while expert reviewers’ open-ended responses were utilized descriptively to establish a recommended refinement and implementation blueprint for future educational usage, rather than modifying the raw AI-generated scenario flow examined in this study. The reporting of this study adheres to the guidelines for health care simulation research as extensions to the CONSORT and STROBE statements [21].

### Ethics

The study commenced after obtaining ethical approval from the Scientific Research Ethics Committee of XXX University. Participants were informed about the purpose of the research and the voluntary nature of their participation. Written informed consent was obtained, and the confidentiality of all data was ensured. The study was conducted in accordance with the Declaration of Helsinki.

### Scenario development process

The development process was designed to create a comprehensive simulation scenario package. The structural components of this package (learning objectives, prebriefing, and debriefing) were grounded in the INACSL Healthcare Simulation Standards of Best Practice™ [22]. For the generation of the specific clinical scenario flow (script) within this package, we utilized the seven-step methodology described by Allgood and Pruitt to systematically leverage Generative AI [23]. These steps include: (1) identifying competencies and objectives, (2) drafting brief scenario scenes, (3) preparing character sketches, (4) adding guiding statements to each scene, (5) organizing and refining the scenes, (6) creating discussion questions, and (7) compiling references. The first three steps were developed based on the input of subject matter experts experienced in orthotic fabrication (Authors blinded for review) and hand therapy (Authors blinded for review), as well as foundational textbooks in the field [24, 25]. Following the completion of these steps, the subject matter experts-derived inputs served as structured prompts for the Generative AI, which was then used to support the drafting and refinement of the remaining scenario components.

#### Step 1

Accordingly, seven learning objectives were formulated. The student listened to the patient’s history and identified motor, sensory, muscle strength, and atrophic findings related to ulnar nerve injury. The student selected an anticlaw orthosis, obtained the required measurements, prepared a template, and transferred it to thermoplastic material. During orthosis application, the student positioned the patient appropriately, verified joint angles, heated the material correctly, and applied it to the hand. The student evaluated the completed orthosis with respect to edge smoothness, pressure areas, range of motion, and functional fit, and made adjustments as needed. Finally, the student provided education to the patient and caregiver regarding orthosis use and scheduled a follow up appointment.

#### Step 2

The scenario structure followed the 4 C framework. Causality emphasized the clinical need for an anticlaw orthosis to manage claw hand deformity resulting from ulnar nerve laceration. Conflict involved the patient experiencing difficulty donning and doffing the orthosis while the caregiver attempted to record the education process using a mobile phone. The characters included the therapist, therapy assistant, patient, and caregiver. Complications included the orthosis heating pan not being pre warmed, resulting in a delay; the caregiver’s frustration with the delay; and redness and pressure over the little finger caused by the orthosis. The scenes were organized in alignment with the predefined learning objectives.

#### Step 3

The number of characters was limited to five or fewer. The scenario characters were described as follows: a 35 year old therapist with ten years of experience in hand rehabilitation who practices empathetically and in an evidence based manner; a 22 year old therapy assistant who is technically competent but occasionally struggles with timing; a 55 year old right hand dominant homemaker as the patient; and her 60 year old husband as the caregiver, who actively participates in the process but may demonstrate impatience during delays or unclear instructions.

**Fig. 1:**
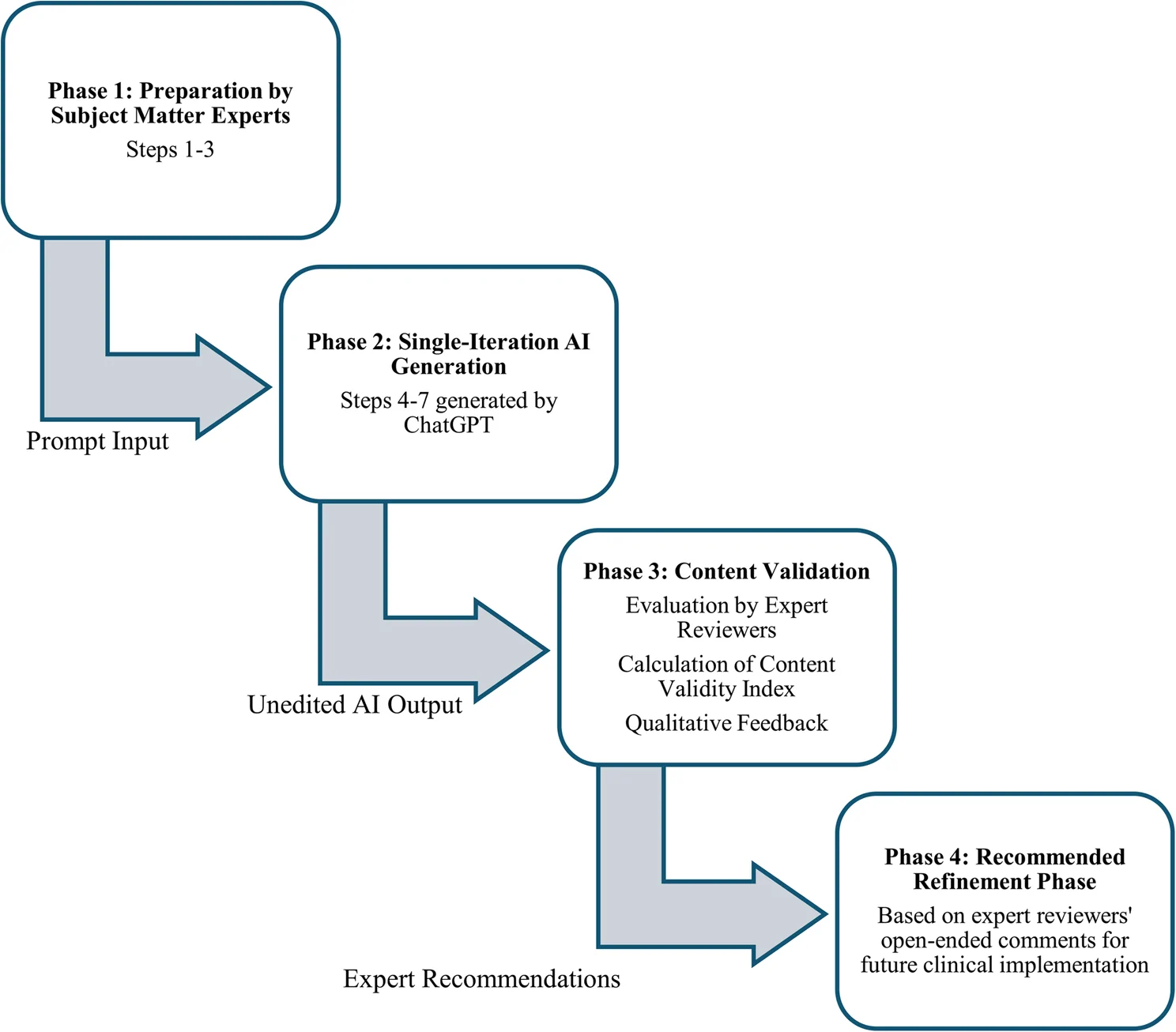
Flowchart of the AI-assisted scenario flow development and content validation process

The involvement of generative AI (ChatGPT) was strictly limited to a single iteration. Steps 1–3 were developed entirely by human subject matter experts without AI assistance. Subsequently, in Steps 4–7, ChatGPT was employed exclusively to draft the scenario narrative, utilizing the subject matter expert outputs as direct prompts. The prebriefing and debriefing components were developed entirely by the subject matter experts without AI assistance. The complete scenario package was then compiled and submitted directly to the expert reviewers via email for content validation (Supplement 1). Thus, the present study evaluates the unedited AI output, reporting its validated elements, followed by a recommended refinement phase (Phase 4) where the expert reviewers’ qualitative feedback is structured as a blueprint for future clinical implementation (Fig. 1).

### Participants

In line with Lynn’s recommendation that CVI assessments should be conducted by a minimum of six expert reviewers [26], the participants of this study consisted of six volunteer expert reviewers who were reached through purposive sampling [27]. Eligibility criteria included professional experience in at least one of the following areas: hand rehabilitation, orthotic fabrication, or simulation based education. The exclusion criteria for the study were defined as having fewer than five years of clinical or academic experience in the relevant field. Additionally, experts who were unable to complete the evaluation within the designated timeframe were considered non-responders, and their data were not included in the final analysis.

### Data collection tools

Data were collected using a Google Forms survey developed by the researchers. The English version of the expert reviewer evaluation form is provided in (Supplement 2). The survey consisted of two sections and was distributed to expert reviewers via email. Participants were given up to one month to complete the evaluation.

The first section collected demographic and professional information, including age, academic title, areas of expertise such as hand rehabilitation, orthotic fabrication, and simulation, and years of professional experience.

The second section asked expert reviewers to evaluate the scenario across five core components: learning objectives, background information, prebriefing, scenario details, and debriefing instructions. Item relevance was rated using a four point Likert type scale ranging from 1 (not relevant) to 4 (highly relevant) [26, 28]. An open ended question at the end of the survey invited additional comments and recommendations regarding scenario strengths and areas for improvement.

### Data analysis

Quantitative data were analyzed using the CVI at both item and scenario levels. Item level CVI values were calculated by dividing the number of expert reviewers assigning ratings of 3 or 4 by the total number of expert reviewers. Scenario level CVI values were calculated by averaging all item level CVI scores. A CVI threshold of 0.80 or higher was considered acceptable, consistent with previous content validity studies [28, 29].

Qualitative feedback obtained from the open-ended questions was not used to alter or manually edit the simulation scenario flow within this study, as the primary objective was to evaluate the content validity of the raw, unedited generative AI output. Instead, in alignment with a qualitative descriptive approach to instrument refinement [30], these open-ended responses were critically reviewed and categorized by a single researcher based on their focus areas to serve as a practical ‘Refinement and Implementation Guide’ (Phase 4) for future implementation.

## Results

A total of six expert reviewers participated in the study. Their demographic and professional characteristics are presented in (Table 1). The distribution of academic titles was as follows: The sample included three professors (*n* = 3, 50%), one associate professor (*n* = 1, 16.7%), and two assistant professors (*n* = 2, 33.3%). The expert reviewers professional experience ranged from 12 to 36 years, and their experience levels were evenly distributed across the categories of 10–20 years, 21–30 years, and 31 + years (each *n* = 2, 33.3%). The areas of expertise of the participants were identified as follows: hand rehabilitation (*n* = 1, 16.7%), orthotics (*n* = 2, 33.3%), nursing education (*n* = 2, 33.3%), and health services management (*n* = 1, 16.7%).

**Table 1:** Demographic and professional characteristics of the expert reviewers

Characteristic	Category	*n*	%
Academic Title	Professor Associate Professor Assistant Professor	3 1 2	50.0 16.7 33.3
Professional Experience	10–20 years 21–30 years 31 + years	2 2 2	33.3 33.3 33.3
Area of Expertise	Hand Rehabilitation Orthotics Nursing Education Health Services Management	1 2 2 1	16.7 33.3 33.3 16.7

*n*  Number of expert reviewers, *%* Percentage

The simulation scenario package was evaluated across five core components: objectives, background information, prebriefing, scenario details (flow), and debriefing instructions. For all scenario items, the item-level content validity index (I-CVI) was calculated to be 1.00. None of the expert reviewers assigned a rating of “not relevant (1)” or “somewhat relevant (2)” to any item. All ratings were clustered at the levels of “quite relevant (3)” or “highly relevant (4)” (Table 2).

**Table 2:** Distribution of expert reviewer ratings by item

Evaluated Item	Rating 3 (Quite Relevant) (*n*, %)	Rating 4 (Highly Relevant) (*n*, %)	I-CVI (Proportion of 3 + 4)
1.Objectives	4 (%66.7)	2 (%33.3)	1.00
2.Background Information	2 (%33.3)	4 (%66.7)	1.00
3. Prebriefing	1 (%16.7)	5 (%83.3)	1.00
4. Scenario Details	3 (%50.0)	3 (%50.0)	1.00
5. Debriefing	2 (%33.3)	4 (%66.7)	1.00
Overall Total (30 ratings)	12 (%40.0)	18 (%60.0)	-

*n*  Number of expert reviewers, *I-CVI*  Item-Level Content Validity Index.

Among the 30 ratings evaluated, 18 (60%) were classified as “highly relevant,” while 12 (40%) were designated as “quite relevant.” Given that all items possessed an I-CVI value of 1.00, the scale-level content validity index (S-CVI), which signifies overall validity, was consequently determined to be 1.00. This finding suggests that the scenario exhibits a high degree of content validity.

To establish the Phase 4 ‘Refinement and Implementation Guide’, the open-ended feedback provided by the expert reviewers was systematically reviewed and organized. These recommendations were grouped by a single researcher into four main categories that highlight the primary areas for future scenario flow optimization.

### Category 1: temporal and laboratory equipment management

To optimize the physical simulation environment, expert reviewers emphasized the importance of detailed equipment specifications to enhance practical clarity and usability:

“To support the upper extremity, a table, appropriate chairs for the participants, a measuring tape for circumference measurements, and basic materials such as a skin-safe marking pen, used for marking excess areas on the orthosis during fitting, could be more explicitly specified to improve clarity during implementation.”

Furthermore, regarding the management of simulation flow, the experts highlighted that structured time planning is essential to maintain the scenario’s temporal realistic pace:

“A time plan indicating approximately how long it should take to complete each scene could be developed.”

### Category 2: patient safety and education

Expert reviewers emphasized the importance of integrating explicit safety precautions to mitigate student and patient anxiety during procedural applications:

“In Scene 4, an explanation could be added to reassure that the patient will not get burned during the application of the heated thermoplastic material.”

Furthermore, regarding patient education, the experts recommended incorporating active verification steps to ensure the patient has accurately processed the provided information:

“After patient education, the patient could be asked to summarize the instructions, followed by feedback to ensure understanding.”

### Category 3: role and communication competencies

Expert reviewers suggested expanding the scenario’s scope to include interprofessional collaboration. Specifically, they recommended enhancing teamwork and professional dialogue:

“The section describing the therapist’s colleague could include an orthotics–prosthetics technician/assistant, allowing professional dialogue and teamwork communication to be emphasized.”

Furthermore, reviewers highlighted the need for more standardized and detailed instructions for simulation participants to improve realism and behavioral consistency:

“A detailed guide could be prepared for the person or student playing the patient role, specifying pain level, anxiety status, and how they should behave in different parts of the scenario.”

In addition to role descriptions, emphasis was placed on integrating essential clinical communication steps into the student’s required competencies:

“The competency section could include steps such as the therapist introducing themselves and informing the patient before the procedure.”

### Category 4: debriefing processes

Expert reviewers also provided recommendations related to feedback and evaluation processes during debriefing:

“In the Final Evaluation stage, a structured feedback technique such as the Plus–Delta Method could be used to help students engage in critical reflection.”

“During debriefing, the discrepancies between what should have been done and what was actually performed in the simulation could be discussed.”

“If available, reviewing the video recording afterward could provide additional feedback.”

“The nonjudgmental feedback approach is highly effective.”

## Discussion

To the best of our knowledge, this study was among the first to examine the content validity of an AI-assisted simulation scenario within occupational therapy education. The findings demonstrated that the AI-assisted simulation scenario package for anticlaw orthosis fabrication achieved high content validity, with all items rated as “quite relevant” or “highly relevant” by expert reviewers. High CVI values across learning objectives, background information, prebriefing instructions, scenario flow, and debriefing components indicated that the scenario content was appropriate for the targeted psychomotor skills when evaluated against established criteria. In addition, qualitative expert reviewer feedback provided critical refinements categorized into four key areas, encompassing temporal and laboratory equipment management, patient safety and education, role and communication competencies, and debriefing processes.

In health professions education, the limitations of conventional teaching methods, particularly in fostering teamwork and problem solving skills, have long been recognized [31]. Simulation based education was increasingly adopted to address these limitations; however, resources focusing specifically on systematic scenario development and validation remained limited [32]. Previous studies reported that validity procedures were frequently overlooked during scenario development and that non systematic approaches were commonly employed [19]. In a review of simulated patient scenarios, Mirza et al. found that scenario development processes were largely based on researcher experience rather than standardized, evidence based methods [33]. Establishing content validity was therefore described as a critical step in determining whether a simulation scenario accurately represented clinical situations and provided appropriate cues to guide learners’ clinical decision making [34]. Recent research examining AI assisted scenario generation highlighted both the potential utility and the limitations of LLM tools such as ChatGPT. Although these tools were reported to generate medically accurate information, inconsistencies related to scenario structure, depth, realism, and the inclusion of essential clinical details were also observed [17]. In the present study, the AI-drafted scenario narrative was embedded within a comprehensive simulation package validated by expert reviewers. This approach effectively addressed common limitations regarding clinical accuracy and structure, demonstrating that Generative AI serves best as a supportive tool within established educational materials rather than a standalone solution. Unlike traditional scenario development, which often relies on informal peer review, the use of AI in this study necessitated a more rigorous and systematic validation through a formal CVI framework to ensure clinical safety and fidelity.

Expert reviewer feedback provided detailed recommendations aimed at enhancing the clinical fidelity and feasibility of the simulation scenario. Suggestions such as specifying materials and equipment in greater detail and establishing a clear time plan for each scene were identified as refinements to support realistic implementation. These findings indicated that, even when high content validity was achieved, expert reviewer input remained essential for strengthening fidelity related aspects of scenario design, particularly in technically demanding psychomotor training. Furthermore, explicitly addressing safety considerations such as burn risk and active verification of patient understanding highlighted the necessity of integrating explicit safety precautions to maintain a secure learning environment. In alignment with these refinements, expert reviewer recommendations regarding role and communication competencies further underscored the value of interprofessional perspectives in simulation design. By incorporating non-technical skills such as therapist self-introduction and patient education into the scenario, the need to embed behavioral competencies alongside technical skill acquisition was clearly highlighted. Previous research supports this approach, showing that structured feedback following simulated encounters facilitates the integration of cognitive, psychomotor, and behavioral domains [35]. Within this context, the addition of an orthotics prosthetics technician role was interpreted as an opportunity to enhance interprofessional communication rather than a limitation. Similarly, providing clearer guidance for the patient role was viewed as a means of extending the scenario’s fidelity beyond a purely technical focus toward a more holistic representation of clinical practice, consistent with Alinier et al.’s finding that well-defined professional roles support realistic team dynamics [36].

Post event facilitated reflection and analysis were consistently emphasized as essential components supporting learning in simulation based education [37]. Experts reviewers’ perspectives regarding the debriefing stage were aligned with the prevailing view that debriefing constitutes a central element of effective simulation practice [38]. Effective feedback was identified as one of the key factors most strongly associated with learning outcomes, and debriefing was recognized as a critical component of simulation based training [39]. Expert reviewers in the present study recommended the use of structured debriefing methods, such as the Plus–Delta approach, to support learners’ critical reflection. This recommendation was consistent with prior findings identifying the Plus–Delta method as a simple and effective strategy for enabling participants to evaluate their own performance [40]. In addition, the optional use of video recordings was highlighted by expert reviewers as a supplementary feedback tool when available, in line with evidence suggesting that video review can facilitate behavior change [41]. Research examining barriers to simulation based learning demonstrated that participants may perceive simulation environments as stressful and may experience concern regarding evaluation by instructors or peers [42]. Prior literature emphasized that the effectiveness of debriefing was contingent upon the establishment of a psychologically safe learning environment in which participants felt valued and respected, as such environments facilitated honest, open, and reflective sharing of experiences [43, 44]. In the present study, expert reviewers’ positive appraisal of a nonjudgmental feedback approach suggested that the debriefing structure aligned with these established principles of psychological safety and reflective learning.

### Limitations

Several limitations should be considered when interpreting the findings of this study. First, the content validity assessment was based on expert reviewer judgment, and the simulation scenario had not yet been pilot tested with occupational therapy students at the time of the study. Therefore, the direct effects of the scenario on learners’ psychomotor performance, learning outcomes, and educational experiences were not examined and remained beyond the scope of this research. This approach was intentionally selected to prioritize review by expert reviewers and content validation prior to learner exposure. Second, the study methodology focused on orthosis fabrication as a specific psychomotor skill domain within occupational therapy education. Although elements related to communication and patient interaction were incorporated into the scenario design, the primary emphasis was placed on technical and procedural competencies. As a result, the findings may have limited applicability to simulation scenarios primarily targeting communication skills, learner affect, or complex psychosocial processes. Finally, the generative AI model used to support scenario development was subject to ongoing updates and version changes, meaning that the findings reflected the performance and characteristics of the model at the time of data collection. The reproducibility of AI assisted scenario drafts across different model versions was not examined. Future studies may therefore explore learner outcomes, perceived fidelity, and user experience associated with AI assisted simulation scenarios, as well as the consistency of AI supported content generation across evolving models and diverse educational contexts.

## Conclusion

This study examined the content validity of a simulation scenario for anticlaw orthosis fabrication developed with the support of generative AI within occupational therapy education. The findings demonstrated that, when guided by a structured development process and review by expert reviewers, an AI assisted simulation scenario could achieve high content validity for a targeted psychomotor skill. Expert reviewer feedback further highlighted the importance of human oversight to ensure clinical fidelity, feasibility, and patient safety. Accordingly, generative AI should be viewed as a supportive tool rather than a stand alone solution in simulation design. This study provides a validated educational resource for orthotic fabrication training and supports the careful integration of AI assisted tools into occupational therapy education.

## Supplementary Information

Supplementary Material 1.

Supplementary Material 2.

## Data Availability

The datasets used and/or analysed during the current study are available from the corresponding author on reasonable request.

## Abbreviations

AI: Artificial Intelligence
CVI: Content Validity Index
I-CVI: Item-Level Content Validity Index
S-CVI: Scale-Level Content Validity Index
INACSL: International Nursing Association for Clinical Simulation and Learning
LLM: Large Language Model
OT: Occupational Therapy
