# Supplementary material for: Development and content validity of an AI-assisted simulation scenario for orthotic fabrication in occupational therapy education
Source: BMC Med Educ. 2026 Jul 29;26:1726. doi: 10.1186/s12909-026-10043-x (PMC13711834; doi:10.1186/s12909-026-10043-x)
Supplement: Supplementary file 1 [file 12909_2026_10043_MOESM1_ESM.docx]

**This document was prepared for expert reviewers.**

**1.Learning Objectives**

The competencies expected to be demonstrated by students within the simulation scenario will be evaluated based on the items listed in the observation form below.

**Anticlaw Orthosis Simulation – Student Observation Form**

Student Name: …………………
Evaluator: …………………
Date: …………………

Each competency will be rated using the following scale: **1 = Insufficient | 2 = Needs Improvement | 3 = Competent | 4 = Excellent**

**1. Therapist Conducts Patient Interview and Evaluation**

- Determines the patient’s interests, goals, occupational performance, environmental conditions, financial resources, family structure, psychosocial status, age, dominant hand, support systems, and profession. [1] [2] [3] [4]
- Identifies when and how the injury occurred and whether surgical intervention was performed. [1] [2] [3] [4]
- Measures ROM of the 4th and 5th MCP, PIP, and DIP joints. [1] [2] [3] [4]
- Evaluates Tinel’s sign and monitors nerve regeneration. [1] [2] [3] [4]
- Observes atrophy in hypothenar, interosseous, and lumbrical muscles. [1] [2] [3] [4]
- Performs manual muscle testing for hypothenar, interosseous, and lumbrical muscles. [1] [2] [3] [4]
- Performs sensory assessment in the ulnar nerve innervation regions. [1] [2] [3] [4]

**2. Determines Orthosis Based on Evaluation Findings**

- Considers the history of ulnar nerve laceration and repair. [1] [2] [3] [4]
- Selects an anticlaw orthosis involving the 4th and 5th fingers. [1] [2] [3] [4]

**3. Takes Measurements from the Patient**

- Accurately measures the proximal phalanx width of the 4th and 5th fingers. [1] [2] [3] [4]
- Measures the proximal phalanx length of the 4th and 5th fingers and the metacarpal circumference of the hand. [1] [2] [3] [4]
- Calculates the measurements determined by the proximal phalanx width and 30% less than the total circumference, and creates a template on A4 paper. [1] [2] [3] [4]
- Transfers the drawn pattern from the A4 paper onto the thermoplastic sheet [1] [2] [3] [4]

**4. Applies the Orthosis to the Patient**

- Places thermoplastic material properly into the heating pan. [1] [2] [3] [4]
- Positions patient seated with elbow in flexion. [1] [2] [3] [4]
- Positions ring and little finger at 30–45° MCP flexion and IP joints in extension. [1] [2] [3] [4]
- Controls joint positioning using a goniometer. [1] [2] [3] [4]
- Applies the heated thermoplastic strip in an figure-of-eight configuration. [1] [2] [3] [4]
- Instructs the patient to maintain position until the orthosis cools. [1] [2] [3] [4]

**5. Evaluates the Finished Orthosis**

- Identifies and corrects sharp or rough edges. [1] [2] [3] [4]
- Ensures orthosis does not restrict wrist, thumb, or index–middle finger movements. [1] [2] [3] [4]
- Ensures no ink or fingerprints are left on the orthosis surface. [1] [2] [3] [4]
- Checks that MCP extension is limited appropriately. [1] [2] [3] [4]
- Evaluates redness/pressure areas and modifies using heat gun if necessary. [1] [2] [3] [4]
- Observes occupational performance using a simulated task. [1] [2] [3] [4]

**6. Provides Patient Education on Orthosis Use and Care**

- Explains the correct usage period (until muscle strength reaches at least Grade 3). [1] [2] [3] [4]
- Advises avoiding heavy lifting, resisted exercise, and repetitive movements. [1] [2] [3] [4]
- Recommends day and night use. [1] [2] [3] [4]
- Explains proper cleaning (warm soapy water; no alcohol). [1] [2] [3] [4]
- Emphasizes avoiding hot water/washing machine. [1] [2] [3] [4]
- Demonstrates donning and doffing techniques; caregiver video recording may be encouraged if needed. [1] [2] [3] [4]

**7. Plans Follow-Up Appointment**

- Schedules follow-up in 3 weeks, considering nerve regeneration timeline. [1] [2] [3] [4]

**8. Observer Notes:**

…………………………………………………………………………………………………………………………………………………………………………………………………..

**2. Background Information For Patient Scenario**

**Patient:**
A 55-year-old right-hand-dominant woman living with her husband. She performs household tasks primarily with her right hand. One month ago, she sustained a laceration while opening a package with a knife, resulting in an ulnar nerve lesion ~5 cm proximal to Guyon’s canal. She underwent end-to-end microsurgical repair and was referred for orthosis fabrication. No systemic disease or allergies. She experiences limitations in daily activities and expresses anxiety about regaining hand function. She is motivated for rehabilitation.

**Caregiver (Husband):**
Supportive but occasionally impatient during treatment instructions.

**Therapist:**
A 35-year-old experienced hand therapist using empathetic communication and evidence-based practice.

**Therapy Assistant:**
Works with therapist, manages heating/preparation of materials; may experience timing-related stress.

**3. Pre-briefing Instructions**

**Purpose of the Scenario**

This simulation is designed to provide experience in evaluation, orthosis decision making, measurement taking, fabrication, fitting, patient/caregiver education, and follow-up planning after an ulnar nerve injury above the wrist.

**Environment and Roles**

The simulation takes place in a clinical rehabilitation setting.
Roles include therapist, patient, caregiver, and therapy assistant.

**Expectations**

- Professional behavior
- Empathetic patient interaction
- Safe and evidence-based clinical decision making
- Learning-focused environment

**Rules**

- Confidentiality is essential
- Respect all roles
- Consult the instructor when questions arise
- Time efficiency is important

**Materials Used**

-Orthosis heating pan

- Low-temperature thermoplastic

- Goniometer

- A4 paper

- Scissors

-Pen

- Adhesive bandage

-Utility knife

- Thermoplastic scissors

- Bucket

- Water

- Towel

-Spatula

- Monofilament

**Evaluation:**
The students’ performance will be assessed using the observation form, and a final debriefing session will be conducted at the end of the scenario.

**4. Scenario Details (Flow)**

**Scene 1 – Patient Interview and Evaluation**

A middle-aged woman enters the therapy room slowly together with her husband. She holds her right hand close to her body; there is hesitation in her steps and a noticeable concern on her face. Her eyes scan the room briefly as she considers where to sit.

Therapist Ayşe stands up, smiles, and speaks with a soft tone:
— *“Welcome. I see a bit of concern on your face; how are you feeling right now?”*

The patient, holding her hand with her other hand, replies:
— *“Yes, I am worried… I’m afraid I won’t be able to use my hand like before.”*

Ayşe nods empathetically:
— *“That is a very understandable feeling. We will manage this process together in the best possible way.”*

She guides the patient to sit and continues the conversation through daily activities:
— *“How does a typical day go for you? Which activities are important in your daily life?”*

The patient sighs and explains:
— *“I am a homemaker. I used to do housework, cooking, mopping… all with my right hand. Now most of it is very difficult.”*

While maintaining eye contact and taking notes, Ayşe transitions to the injury:
— *“How did this situation begin; how did the injury occur?”*

The patient gives a brief explanation:
— *“I cut just below my wrist while opening a package with a knife in the kitchen. They said the nerve was cut. Then I had surgery.”*

Ayşe glances at the surgical note in the file and shares the information with the patient:
— *“Your notes indicate that the injury occurred about 5 cm proximal to Guyon’s canal and that an end-to-end repair was performed. You were referred for orthosis fabrication approximately one month after the surgery. This is important information for us.”*

After a short pause, Ayşe explains the plan:
— *“Now I will take a detailed history from you. Then I will need to perform some evaluations. This will help me understand your condition and fabricate the most appropriate orthosis for you.”*

The patient nods in agreement:
— *“Okay, I’m ready.”*

The therapist completes the detailed history and performs the necessary assessments.

**Scene 2 – Evaluation Data and Orthosis Decision**

After taking the history and completing the evaluation, Therapist Ayşe reviews her notes. After thinking for a moment, she turns to the patient:
— *“I have reviewed the information related to your condition. Based on this, I need to choose the most appropriate orthosis. This decision is very important for your nerve injury, your needs during daily activities, and your recovery process.”*

The patient asks curiously:
— *“Will the orthosis completely restrict my hand movements? Will cooking become even harder?”*

Ayşe responds in a reassuring tone:
— *“We will also evaluate how you use your hand in daily life with the orthosis. Our goal is not to restrict you; it is to support your recovery and prevent deformity.”*

She continues, considering the clinical reasoning:
— *“There are motor and sensory deficits. In this case, we need to fabricate an anticlaw orthosis including the 4th and 5th fingers, carefully considering pressure points.”*

After taking a brief note, she says to the patient:
— *“I believe this orthosis will be the most suitable option for you. The orthosis we will make will help preserve the function of your fingers and prevent deformity.”*

**Scene 3 – Taking Measurements**

Therapist Ayşe prepares to take measurements and explains to the patient:
— *“Now I need to take some measurements from your hand in order to prepare your orthosis. This procedure will be quick and will not hurt.”*

The patient asks with hesitation:
— *“My hand is very sensitive; will it hurt during the measurement?”*

Ayşe smiles and answers:
— *“Don’t worry, I will only be using a measuring tape. It is a completely safe and painless process for you.”*

Ayşe slowly reaches for the patient’s hand while speaking in a calming tone:
— *“Keeping your hand in this position will be enough. I will take the measurements carefully.”*

She takes the measurements with a tape measure, records the values, and completes the process.

The patient waits quietly during the measurement, occasionally watching the process curiously. After finishing, Ayşe turns to her:
— *“Very good, we have our measurements. Now we will move on to preparing your orthosis.”*

**Scene 4 – Application of the Orthosis**

After completing the measurements, Ayşe turns to the patient:
— *“Now we will prepare your orthosis. This will take some time; I just need you to stay in a comfortable position.”*

The patient nods in acceptance.

Ayşe seats the patient and ensures proper positioning. She adds:
— *“It is very important that you maintain this position, we will begin applying the orthosis shortly.”*

Meanwhile, the young technician assisting the therapist is preparing the materials. They need to wait for the orthosis heating pan to reach the correct temperature, as it was just turned on, and the water takes time to heat. During the wait, the patient remains patient, but the caregiver grumbles:
— *“Does it really have to take this long? Couldn’t this have been prepared earlier?”*

Ayşe explains calmly:
— *“You are right, we are waiting a little. But it is very important that the material reaches the correct temperature. That way the orthosis will be both safe and comfortable to wear.”*

Soon, the thermoplastic strip becomes transparent. The therapy assistant carefully removes it with a spatula and places it on a towel. Ayşe waits for the initial heat to subside, then applies it to the patient’s hand.

— *“Please hold this position for a few seconds. You need to remain still until the material cools.”*

The patient nods and keeps the hand still. Ayşe monitors the cooling process closely.

**Scene 5 – Checking the Orthosis**

Therapist Ayşe examines the orthosis carefully. While feeling the edges of the strip, she murmurs to herself:
— *“The edges need to be smooth… function must not be restricted.”*

She checks whether the orthosis limits hand movement. She then says to the patient:
— *“Now, could you slowly open and close your hand?”*

The patient moves her fingers. Ayşe observes attentively:
— *“Good, the orthosis is effectively limiting MCP extension.”*

During the check, she notices a small detail and corrects it. Then she explains:
— *“Now I would like you to stay here for a moment. During this time, we will observe how your skin responds.”*

After a short while, the patient points to her little finger:
— *“I feel some pressure near my 5th finger, it is slightly red.”*

Ayşe examines it carefully, identifies the pressure area, and performs a localized adjustment:
— *“I will reshape this area so that it feels more comfortable.”*

After the adjustment, she asks:
— *“How does it feel now?”*

The patient smiles slightly:
— *“Better, it’s not uncomfortable anymore.”*

Ayşe refers back to the functional evaluation and hands the patient a plate and spoon:
— *“Now I would like to see how you use your orthosis in one of your daily activities. Let’s simulate”*

The patient performs the activity while Ayşe observes closely and takes notes.

**Scene 6 – Education on Orthosis Use and Care**

After confirming the final fit, Ayşe turns to the patient:
— *“Now I will explain how you should use the orthosis, how to care for it, and what you should pay attention to.”*

The patient asks:
— *“How long do I need to wear it?”*

Ayşe responds clearly.

The patient's husband interrupts:
— *“So will it cause any problems if she wears it while doing daily tasks, like cleaning?”*

Ayşe shakes her head:
— *“No, but there are some things you need to avoid. You should avoid heavy lifting, resisted exercises, and forceful or repetitive movements. The orthosis will protect you, but overloading your hand can negatively affect the healing process.”*

She then provides instructions for orthosis care.

The patient appears unsure:
— *“But… I’m not sure I understood how to put it on and take it off.”*

Ayşe smiles:
— *“Don’t worry, we will practice together. Your husband can also record it, so you can watch it at home if you forget.”*

The caregiver takes out his phone and begins recording. Ayşe demonstrates the donning and doffing technique step-by-step.

The patient tries several times and eventually does it correctly:
— *“Okay, now I understand.”*

**Scene 7 – Scheduling the Next Session**

After finishing the education session, Ayşe turns to the patient:
— *“We made very good progress today. Lastly, let’s schedule our next appointment.”*

She continues in a reassuring tone:
— *“Nerve regeneration is a process that takes time. We will set a date to reassess your hand and the effectiveness of the orthosis.”*

The patient nods in agreement.

The caregiver adds:
— *“We will arrange our schedule according to that date.”*

Ayşe smiles, notes the appointment, and concludes the session:
— *“Great. Until then, it is very important that you follow the instructions I gave you. If you experience any problems, do not hesitate to contact us.”*

The patient and her husband thank her and leave the room.

**5.Debriefing / Final Reflection Guidelines**

**Purpose**

To discuss experiences, evaluate learning outcomes, identify strengths and areas for improvement.

**Process**

- Students gather in a circle.
- Instructor facilitates discussion in a supportive, non-judgmental environment.

**Discussion Questions**

**Experience & Reflection**

1.What are your thoughts regarding the simulation scenario?

2.How did this experience make you feel?

**Analysis**
3. How well did the scenario reflect clinical reality and curriculum expectations?
4. What skills or problem-solving strategies did you apply?

**Development**
5. What were the strengths of this scenario?
6. What aspects could be improved?
7. Any additional comments?

**Closure**

Instructor summarizes key learning points (patient safety, communication, correct technique, clinical reasoning), and thanks participants.
