# Supplementary material for: Development and content validity of an AI-assisted simulation scenario for orthotic fabrication in occupational therapy education
Source: BMC Med Educ. 2026 Jul 29;26:1726. doi: 10.1186/s12909-026-10043-x (PMC13711834; doi:10.1186/s12909-026-10043-x)
Supplement: Supplementary file 2 [file 12909_2026_10043_MOESM2_ESM.docx]

**Expert Reviewers Evaluation Form for the Orthotic Fabrication Simulation Scenario Package**

Description

This form was developed to evaluate the content validity of the Anticlaw Orthosis Fabrication Simulation Scenario Package used in occupational therapy education. Expert reviewers are asked to assess the relevance and clarity of each core component of the simulation scenario.

Instructions

Please rate each item independently based on its relevance to assessing student competency in orthotic fabrication within occupational therapy education. Use the 4-point scale below.

Rating Scale

1 = Not Relevant

2 = Somewhat Relevant

3 = Quite Relevant

4 = Highly Relevant

**Section 1. Professional Information**

1. Age: __________

2. Academic Title: __________

3. Years of Professional Experience: __________

4. Area of Expertise: __________

**Section 2. Content Validity Evaluation**

**1. Learning Objectives**

The learning objectives are appropriate and clearly defined for evaluating student competency in orthotic fabrication.

[1] Not Relevant [2] Somewhat Relevant [3] Quite Relevant [4] Highly Relevant

**2. Background Information**

The background information provided for the patient scenario is realistic and sufficient.

[1] Not Relevant [2] Somewhat Relevant [3] Quite Relevant [4] Highly Relevant

**3. Prebriefing Instructions**

The prebriefing instructions are clear and adequately prepare students for the simulation.

[1] Not Relevant [2] Somewhat Relevant [3] Quite Relevant [4] Highly Relevant

**4. Scenario Details (Flow)**

The sequence of the scenario is logical and consistent with clinical orthotic fabrication practice.

[1] Not Relevant [2] Somewhat Relevant [3] Quite Relevant [4] Highly Relevant

**5. Debriefing Instructions**

The debriefing questions and guidance support reflection and consolidation of learning outcomes.

[1] Not Relevant [2] Somewhat Relevant [3] Quite Relevant [4] Highly Relevant

**Section 3. Open-Ended Feedback**

Please provide any comments or suggestions regarding the simulation scenario package, including strengths or areas for improvement:
